# miR-19a contributes to gefitinib resistance and epithelial mesenchymal transition in non-small cell lung cancer cells by targeting c-Met

**DOI:** 10.1038/s41598-017-01153-0

**Published:** 2017-06-07

**Authors:** Xiaonian Cao, Senyan Lai, Fayong Hu, Guodong Li, Guihua Wang, Xuelai Luo, Xiangning Fu, Junbo Hu

**Affiliations:** 1 Department of Thoracic Surgery, Tongji Hospital, Tongji Medical College, Huazhong University of Science and Technology, Wuhan, 430030 China; 2 Department of Gastrointestinal Surgery Center, Tongji Hospital, Tongji Medical College, Huazhong University of Science and Technology, Wuhan, 430030 China; 3 Cancer Research Institute, Tongji Hospital, Tongji Medical College, Huazhong University of Science and Technology, Wuhan, 430030 China

## Abstract

Gefitinib, an epidermal growth factor receptor (EGFR) tyrosine kinase inhibitor, is used as a first-line treatment for advanced non-small cell lung cancer (NSCLC). However, most NSCLC patients inevitably develop gefitinib resistance, and the mechanisms underlying this resistance are not fully understood. In this study, we show that miR-19a is significantly down-regulated in gefitinib-resistant NSCLC cell lines compared with gefitinib-sensitive cell lines. In addition, the down-regulation of miR-19a suppressed the expression of epithelial markers but induced the expression levels of mesenchymal markers. A mechanistic analysis revealed that miR-19a regulated c-Met expression by directly targeting the c-Met 3′UTR. Overexpression of miR-19a decreased c-Met expression and re-sensitized gefitinib-resistant NSCLC cells *in vitro* and *in vivo*. Consistent with the *in vitro* findings, the miR-19a serum level was significantly decreased in NSCLC patients with acquired gefitinib resistance compared with the level observed prior to the acquisition of resistance in each patient, indicating that miR-19a expression may be a valuable biomarker for the prediction of acquired gefitinib resistance in a clinical setting. Our data demonstrate that the miR-19a/c-Met pathway plays a critical role in acquired resistance to gefitinib and that the manipulation of miR-19a might provide a therapeutic strategy for overcoming acquired gefitinib resistance.

## Introduction

Lung cancer is the leading cause of cancer-related mortality, accounting for nearly 1.6 million deaths per year^1^. The most common type of lung cancer is non-small cell lung cancer (NSCLC), which comprises approximately 80% of all lung cancer cases. Several genetic alterations in NSCLC, including KRAS gene mutations, EGFR gene mutations, and EML4-ALK rearrangements, have been identified. Of these changes, EGFR gene mutations are found in approximately 10–28% of NSCLC cases and are common in women and non-smokers in the East Asian population^2^.

Most EGFR mutations occur within the kinase domain, leading to the ligand-independent activation of EGFR signaling^3, 4^. Gefitinib, a common tyrosine kinase inhibitor (TKI), has been approved for patients harboring exon 19 deletions or exon 21 (Leu858Arg) substitution EGFR mutations^5, 6^. This drug improves response rates, delays disease progression, and most importantly, increases overall survival compared with platinum-based combination chemotherapy. However, over the course of therapy, many patients experience resistance to TKIs^7, 8^. Currently, several mechanisms of gefitinib resistance have been proposed, including secondary mutations of the EGFR gene at 20 exon (T790M), amplification of c-Met, activation of AXL, activation of EMT, and up-regulation of IGF-1R signaling^2, 9–11^.

MicroRNAs (miRNAs) are a class of small non-coding RNAs that bind to specific sequences in the 3′ untranslated region (3′UTR) of target genes, resulting in degradation of mRNA and/or inhibition of translation^12, 13^. A growing body of evidence indicates that miRNAs play an important role in various biological progresses, including cancer cell proliferation, metabolism development, migration, invasion, differentiation, and drug resistance^13–16^. Recently, several studies have reported that miR-19a plays a complex role in NSCLC cells, and its expression correlates with a worse prognosis in NSCLC patients^17, 18^. Furthermore, Flamant *et al*. reported that miR-19a is a candidate that responds to imatinib mesylate in patients with chronic myeloid leukemia (CML)^19^, indicating that miR-19a may play an important role in the development of resistance to TKIs.

In this study, we showed that the serum level of miR-19a was significantly decreased in patients who developed resistance to gefitinib over the course of gefitinib treatment. Down-regulation of miR-19a expression in gefitinib-sensitive NSCLC cell lines led to gefitinib resistance and EMT. The overexpression of miR-19a in gefitinib-resistant NSCLC cell lines re-sensitized these cell lines to gefitinib. We found that miR-19a contributes to gefitinib resistance and EMT by directly targeting c-Met expression. Taken together, our findings provide a rationale for the use of miR-19a as a predictive biomarker for gefitinib sensitivity and provide a potential therapeutic strategy for overcoming gefitinib resistance by targeting miR-19a expression.

## Materials and Methods

### Ethics statement

All experimental methods in the current study were approved by the research committee of Tongji Hospital at the Tongji Medical College of the Huazhong University of Science and Technology. The study was performed in accordance with the approved guidelines by the Tongji Hospital Ethics Committee. All patients provided written informed consent. All animal studies were performed in accordance with the approved guidelines by the Tongji Hospital Ethics Committee.

### Cell culture and antibodies

The human NSCLC cells (HCC827, H1975, A549, Pc9 and Pc9 GR) were maintained in RPMI-1640 containing 10% fetal bovine serum (FBS). To establish the Pc9 GR cell line, Pc9 cells were cultured, and an appropriate dose of gefitinib (0.1 μM) was added. Then, the dead cells were removed by washing, and living cells were retained and cultured in medium containing gefitinib. The gefitinib concentration was gradually increased. When the concentration of gefitinib reached 5 μM, the surviving Pc9 cells were identified as Pc9 GR cells. A549 cells were also treated with gefitinib before transfected with miR-19a and detected its function on migration ability and EMT.

The following primary antibodies were used in this study: GAPDH (Santa Cruz, sc-32233); p-Erk1/2 (Cell Signaling, 4370 S); Erk1/2 (Cell Signaling, 4695 S); p-Ake^Ser473^ (Cell Signaling, 4060 S); Akt (Cell Signaling, 4691 S); c-Met (Abcom, ab51067); E-cadherin (Cell Signaling, 3195 S); Vimentin (Cell Signaling, 5741 S); and N-cadherin (Cell Signaling, 13116 S).

### miRNA, RNA interference and the lentiviral system

An miR-19a mimic (miR-19a), an miR-19a negative control (miR-NC), an miR-19a inhibitor (Inh-19a), an miR-19a inhibitor negative control (Inh-NC), siRNA duplexes targeting the c-Met gene (si-Met) and an siRNA negative control (si-NC) were purchased from RiboBio (RiboBio Co., Guangzhou, China). The RNA oligonucleotides were transfected using Lipofectamine 2000 (Invitrogen, Carlsbad, CA, USA), and the medium was replaced 6 h after transfection. Final concentrations of 100 nM miR-19a, 100 nM Inh-19a and 100 nM si-Met were used, and the expression levels of miR-19a and c-Met were quantified after 48 h.

The lentivirus carrying the miR-19a precursor sequence (lv-19a), the empty lentivirus (lv-NC), the antisense miR-19a precursor sequence (anti-19a) and the empty lentivirus (anti-NC) were purchased from (Genechem Co. LTD Shanghai, China). Lv-19a or anti-19a was added to A549 cells (MOI = 100), and a single clone was selected. miR-19a expression was examined in each cell line.

### RNA isolation and real-time PCR assay

Total mRNA was extracted using TRIzol reagent (Invitrogen, Carlsbad, CA, USA), and reverse transcription was performed using an RT-PCR KIT (Fermentas). Real-time PCR was conducted on an iQ5 Multi-color Real-Time PCR Detection System (Bio-Rad, Hercules, CA, USA) using SYBR Green Real-time PCR Master Mix (TOYOBO, Shanghai, China). The PCRs consisted of 3 min at 95 °C followed by 40 cycles of denaturation for 15 s at 95 °C, annealing for 30 s at 60 °C and a primer extension for 40 s at 68 °C. The relative quantity of miR-19a was normalized to U6, and that of c-Met was normalized to GAPDH according to the following equation: RQ = 2^^−ΔΔCT^.

To detect miR-19a in serum samples, total RNA was extracted from the serum using the miRNeasy mini kit (QIAGEN). Specific reverse-transcription primers for miR-19a and U6 were purchased from RIBO BIO Company (Guangzhou, China). miR-19a expression was detected using SYBR Green Real-time PCR Master Mix and calculated based on the equation RQ = 2^^−ΔΔCT^. All primer sequences are shown in Supplemental Table 1.

### Cell growth assay

The cells were cultured in 96-well plates containing 100 µl of medium before being transfected with miR-19a or Inh-19a. After 24 h, different concentrations of gefitinib were added, and the cells were incubated for another 72 h. Ten microliters of CCK8 was then added to each well, and the absorbance was measured at 450 nm after 1 h. The relative cell viability rate was calculated, and all experiments were performed in triplicate.

### Western blot analysis

Western blot analysis was performed as described previously^20^. Briefly, the cells were treated and collected, and the total cell lysate was denatured and resolved on SDS-polyacrylamide gels before being transferred to polyvinylidene difluoride membranes. After blocking in 5% skim milk, the membranes were probed with primary antibodies followed by horseradish peroxidase-linked secondary antibodies. The membrane was visualized using electrochemiluminescence (ECL; Pierce Biotechnology, Rockford, IL, USA) and exposed to the chemiluminescence instrument.

### Plasmid construction and luciferase activity assay

The 3′UTR sequence of c-Met was cloned into the pGL3-basic vector. The 3′UTR sequence of c-Met was amplified with a specific primer (Supplemental Table 1) and inserted into the pGL3-basic vector using restriction enzymes. The binding sites of miR-19a and c-Met 3′UTR were predicted using the website http://www.microrna.org/microrna/home.do and the miRanda software. The mutation vector of c-Met 3′UTR was constructed using a Fast Mutagenesis System Kit (TRANSGEN BIOTECH, Beijing, China).

The luciferase activity assay was performed using the Dual-Luciferase Reporter Assay System (Promega, Madison, WI, USA) according to the manufacturer’s instructions. Briefly, HEK293A cells of approximately 80% confluence were seeded in 24-well plates. For the c-Met 3′UTR luciferase reporter assay, 100 ng of wild type or mutant luciferase reporter plasmid was co-transfected with 100 nM miR-19a or 100 nM miR-NC (Inh-19a or Inh-NC) into HEK293A cells in a 24-well plate using Lipofectamine 2000. The luciferase activity assay was examined 48 h after transfection using the Dual-Luciferase Assay System. The firefly luciferase activity was normalized to the corresponding Renilla luciferase activity. All experiments were performed in triplicate.

### Cell migration assay

A549 cells were cultured and transfected with miR-19a or miR-NC. After 24 h, the cells were trypsinized, and 10^4^ cells were resuspended in serum-free medium and placed in the upper chamber. The lower chamber contained 10% FBS, which served as a chemoattractant. The cells were cultured for 12 h, and non-migrating cells were then removed by washing. The migrated cells were washed twice with PBS, fixed in 100% methanol, and stained with crystal violet. The stained cells were viewed under a microscope (200x magnification), and the number of migrated cells was counted in five random fields.

### Clinical sample collection

Procedures involving human subjects were approved by the Huazhong University of Science and Technology Ethics Committee, and a formal form was explained to each subject to ensure full understanding and consent. The blood samples were obtained from lung cancer patients harboring EGFR mutations at 19 or 21 exon who were administered gefitinib for treatment (pre-resistance). Patients treated with gefitinib ultimately developed drug resistance (identified by Computerized Tomographic Scanning), and their blood samples were obtained at this point (post-resistance). The miR-19a expression in each sample was examined by real-time PCR.

### Animal studies

All animal studies were performed in accordance with the approved guidelines by the Tongji Hospital Ethics Committee. The cells were cultured, collected, washed and resuspended culture medium (approximately 1 × 10^7^/ml) and then subcutaneously injected into athymic nude mice. After each tumor had reached a macroscopic size, gefitinib (100 mg/kg) was orally administered to each mouse every other day, and each tumor was measured. After 3 weeks, the mice were sacrificed, the tumors were collected, and the tumor weights were measured. The tumor volume was calculated using the following the formula: volume = (length × width^2^)/2, and a tumor growth curve was generated.

### Statistical analyses

All statistical analyses were performed using the SPSS 10.0 (SPSS Japan Inc., Tokyo, Japan) statistical software package. The means ± sd were calculated, and a two-tailed Student’s t-test was performed using the data analysis tools provided by the software. In all cases, p < 0.05 was considered to indicate significant differences.

## Results

### Reduction of miR-19a in gefitinib-resistant NSCLC patients and cell lines

**Figure 1:**
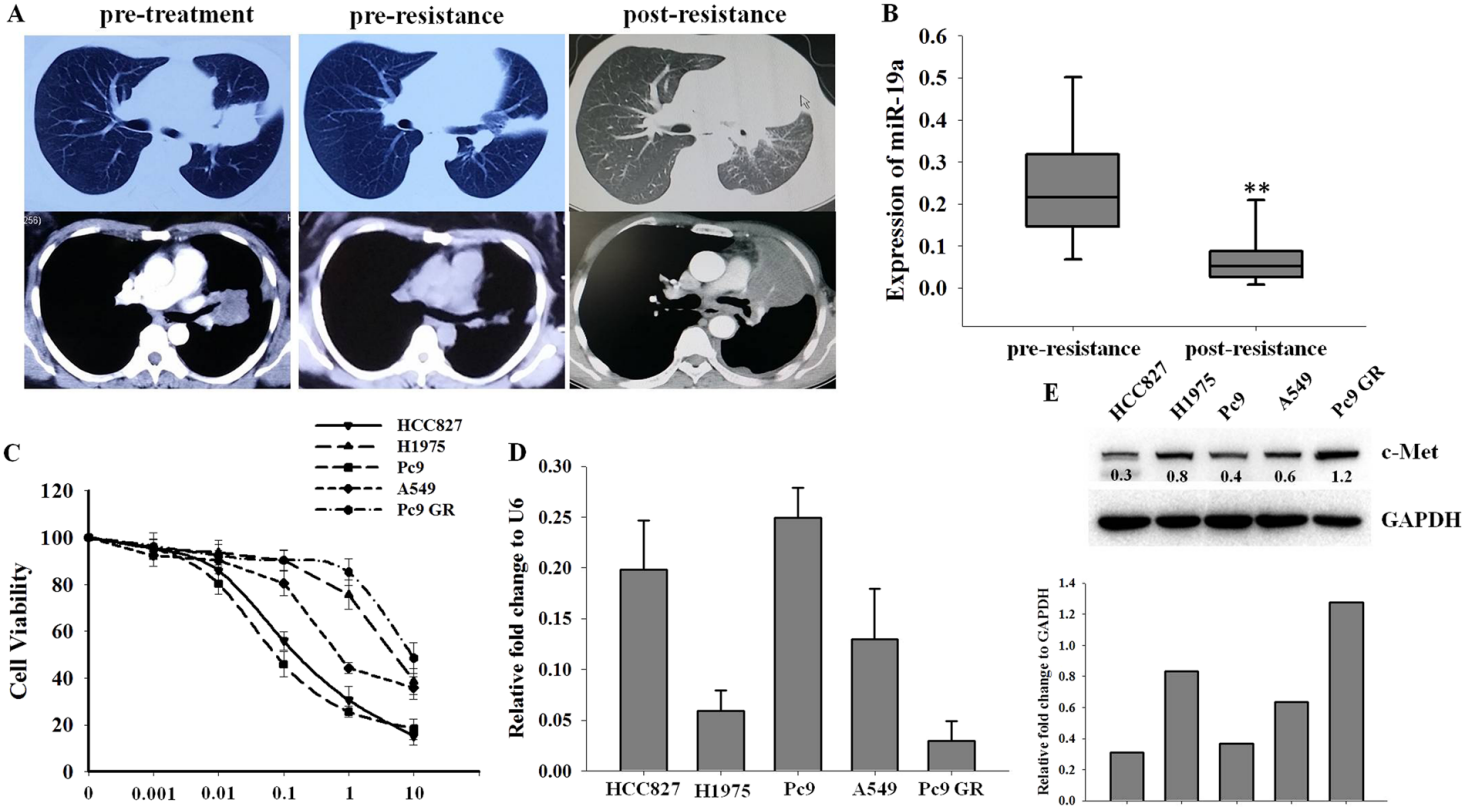
miR-19a expression in NSCLC patient serum samples and cell lines (**A**). Patients were treated with gefitinib and examined monthly with Computerized Tomographic Scanning (CT). Patients were sensitive to gefitinib at the initial stage and became resistant to gefitinib, which was detected by CT; (**B**) The expression of miR-19a was detected in patient serum samples. Serum was obtained from fifteen patients prior to oral gefitinib treatment (pre-resistance) and after they had developed gefitinib resistance (post-resistance). Total RNA was extracted from the serum using the miRNeasy mini kit (QIAGEN), and the miR-19a level was measured by real-time PCR (**p < 0.01); (**C**) NSCLC cell viability was measured. HCC827, H1975, A549, Pc9 and Pc9 GR cells were cultured in 96-well plates and treated with different doses of gefitinib for 72 h before measuring cell viability with CCK8; (**D**) HCC827, H1975, A549, Pc9 and Pc9 GR cells were cultured and harvested; the miR-19a level was measured by real-time PCR, and its relative fold change was compared to that of U6; HCC827, H1975, A549, Pc9 and Pc9 GR cells were cultured and harvested, the expression levels of miR-19a (**D**) and c-Met (**E**, top) were detected, and the expression of c-Met was quantified with GAPDH (**E**, bottom).

miR-19a was previously reported as a candidate that responds to imatinib mesylate in patients with CML, indicating that miR-19a may play an important role in TKI resistance^19^. To investigate the roles of miR-19a in the development of TKI resistance in NSCLC, we first compared the serum expression of miR-19a in patients who initially harbored TKI-sensitive EGFR mutations to the serum level in the same individual after they had acquired resistance to gefitinib. Patients with gefitinib resistance were identified by computerized tomographic scanning over the course of gefitinib treatment (Fig. 1A). Fifteen patients were recruited to our study, and miR-19a expression significantly decreased when patients acquired gefitinib resistance compared with the pre-resistance levels (Fig. 1B). Next, we examined miR-19a expression in five NSCLC cell lines and its relationship with gefitinib resistance. As shown in Fig. 1C, Pc9 GR and H1975 cells were resistant to gefitinib compared with Pc9 and HCC827 cells. Interestingly, miR-19a expression was significantly lower in Pc9 GR and H1975 cells than in Pc9 and HCC827 cells (Fig. 1D). Moreover, miR-19a expression negatively correlated with c-Met expression (Fig. 1E). Our results indicated that miR-19a may be involved in the development of gefitinib resistance in NSCLC.

### Overexpression of miR-19a reverses gefitinib resistance *in vitro*

**Figure 2:**
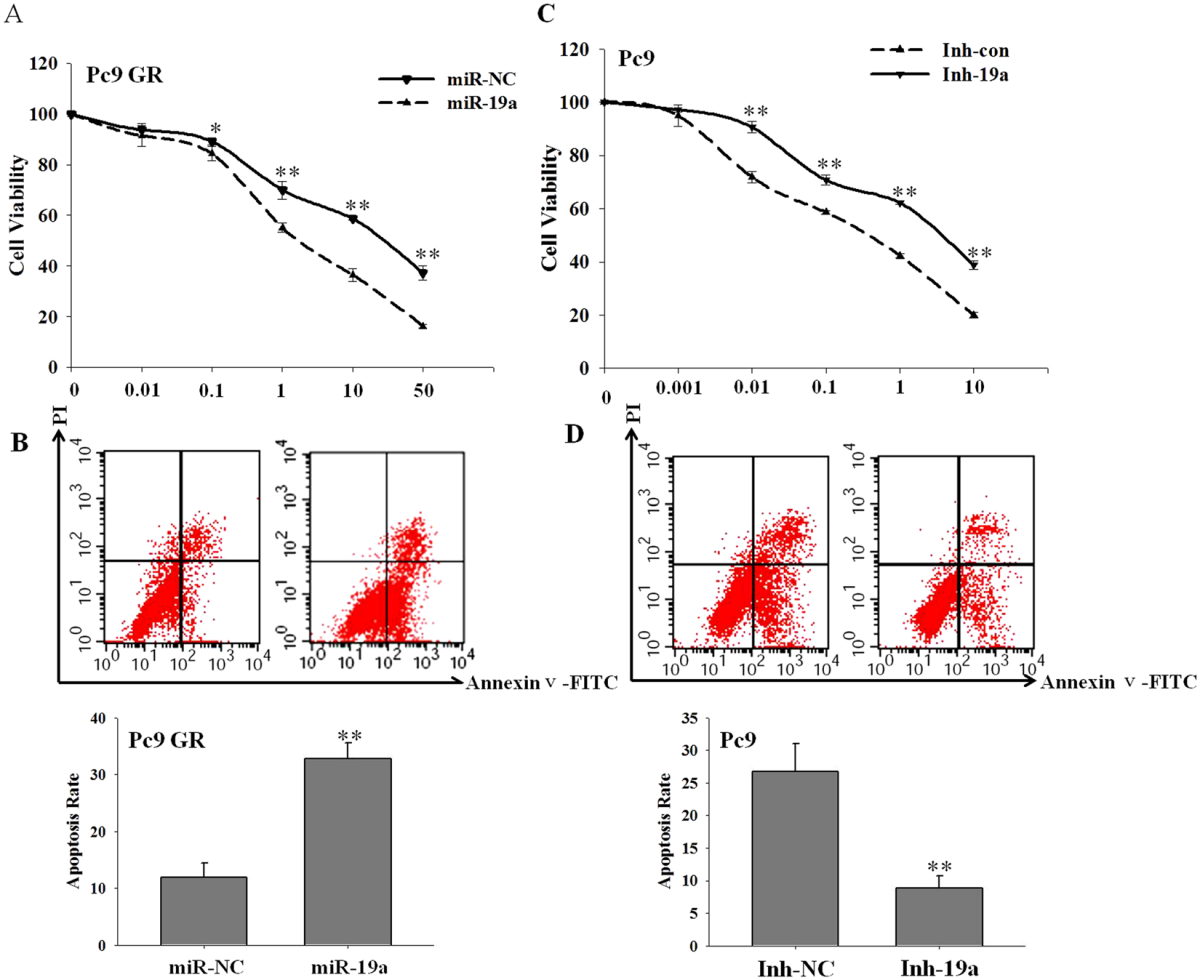
miR-19a overexpression attenuates gefitinib resistance in NSCLC cells (**A**). Pc9 GR (3000/plate) cells were transfected with miR-19a mimics (miR-19a) or the negative control (miR-NC) at a final concentration of 100 nM. The cells were then treated with different doses of gefitinib for 72 h, and viability was assessed using CCK8; (**B**) Pc9 GR cells were transfected with miR-19a or miR-NC and treated with 5 μM gefitinib, and apoptosis was then measured by Annexin V-PI after 48 h; (**C**) Pc9 cells (3000/plate) were transfected with miR-19a inhibitor (Inh-19a) or the negative control (Inh-NC) at a final concentration of 100 nM before being treated with different doses of gefitinib for 72 h and measuring cell viability using CCK8; (**D**) Pc9 cells were transfected with Inh-19a or Inh-NC and treated with 0.1 μM gefitinib before measuring apoptosis by Annexin V-PI after 48 h. (The results above were reproducible in three independent experiments, **p < 0.01).

To confirm that miR-19a contributes to gefitinib resistance in NSCLC cells, we used gain- and loss-of-function approaches in a series of experiments. First, we overexpressed miR-19a by transfecting its mimic in Pc9 GR cells (Fig. S4A) and examined the response of Pc9 GR cell to gefitinib. As shown in Fig. 2A and B, miR-19a overexpression inhibited cell proliferation (Fig. 2A) and increased cell apoptosis (Fig. 2B) in response to gefitinib treatment compared with the cells transfected with mimic control. The down-regulation of miR-19a in Pc9 cells by transfecting cells with miR-19a inhibitor (Fig. S4B) promoted cell proliferation (Fig. 2C) and decreased cell apoptosis (Fig. 2D) upon gefitinib treatment. Similar results were observed in A549 cells (Fig. S1A–D). Thus, our data suggest that miR-19a plays a critical role in the establishment of gefitinib resistance.

### miR-19a contributes to epithelial-mesenchymal transition in NSCLC cells

**Figure 3:**
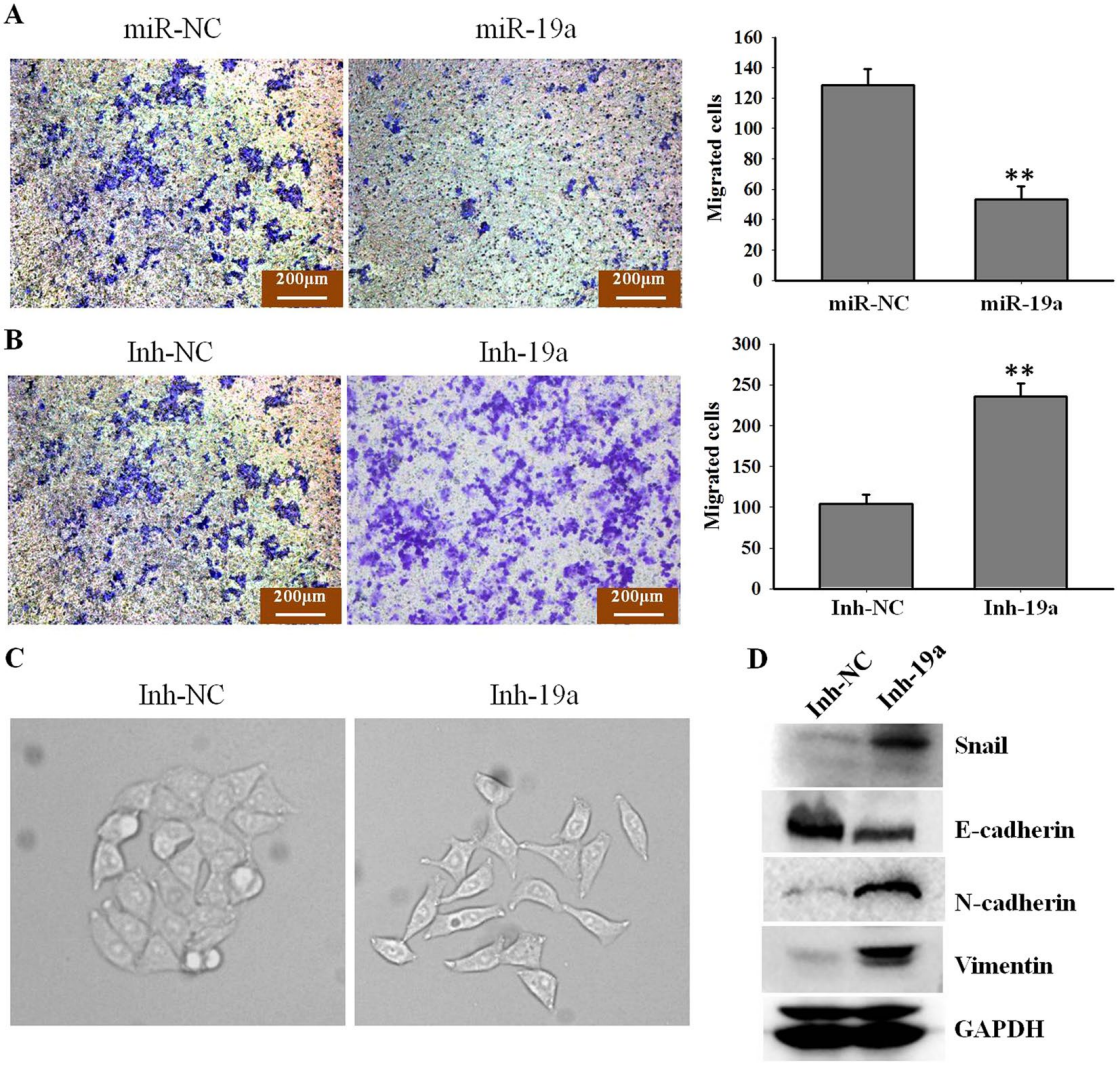
miR-19a contributes to EMT in NSCLC cells (**A**). A549 cells were treated with gefitinib (5 μM) and then transfected with miR-19a or miR-NC; after 24 h, cell migration was detected with a transwell assay. Migrated A549 cells were stained with crystal violet (left); miR-19a overexpression significantly decreased cell migration; quantified data are shown on the right. **p < 0.01; (**B**) A549 cells were treated with gefitinib (5 μM) and then transfected with Inh-19a or Inh-NC; after 24 h, cell migration was detected with a transwell assay, and migrated A549 cells were stained with crystal violet (left); miR-19a down-regulation significantly increased cell migration; quantified data are shown on the right. **p < 0.01; (**C**) A549 cells were treated with gefitinib (5 μM) and then transfected with Inh-19a or Inh-NC; the cell morphology is shown. The Inh-19a group exhibited spindle-shaped cells and loss of cell-cell contact, which suggested EMT; (**D**) A549 cells were treated with gefitinib (5 μM) and then transfected with Inh-19a or Inh-NC, and EMT pathway markers were detected by western blotting.

Previous studies have reported a correlation between gefitinib resistance and the epithelial-mesenchymal transition (EMT)^21, 22^. Therefore, we examined the involvement of miR-19a in EMT. Based on this, we treated A549 cells with gefitinib and then detected miR-19a function on cell migration ability and EMT. A549 cells were transfected with miR-19a mimic or inhibitor, and miR-19a expression (Fig. S4C,D) and migration ability was detected in each group. Our results showed that miR-19a overexpression inhibited the ability of A549 cells to migrate (Fig. 3A), whereas reducing miR-19a expression increased cell migration (Fig. 3B). Furthermore, A549 cells transfected with Inh-19a displayed a spindle shape under a microscope and a loss of cell-cell contact (Fig. 3C), indicating that EMT had occurred (Fig. 3C) upon the decrease in miR-19a expression. To confirm the role of miR-19a in EMT, epithelial and mesenchymal markers were evaluated, and the results indicated that the down-regulation of miR-19a inhibited epithelial markers, whereas mesenchymal markers were increased (Fig. 3D). Collectively, our data suggested that decreased miR-19a expression may contribute to NSCLC cell metastasis by increasing cell mobility and migration and promoting EMT.

### miR-19a decreases c-Met expression and blocks its downstream pathway

**Figure 4:**
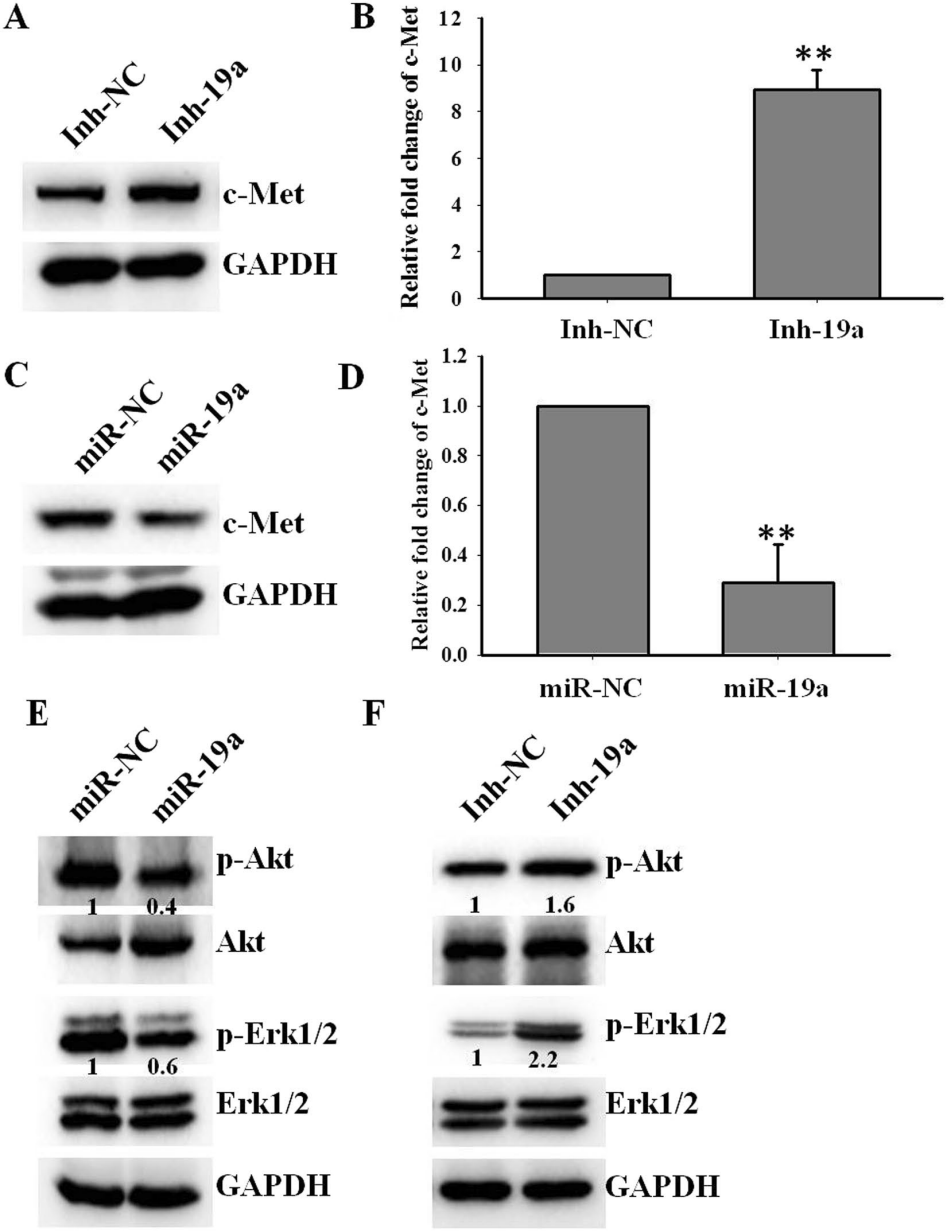
miR-19a decreases c-Met expression and blocks its downstream pathway (**A**,**B**). Down-regulating miR-19a increased the c-Met protein and mRNA levels. Pc9 cells were treated with gefitinib (0.1 μM) and then transfected with Inh-19a or Inh-NC, after 48 h, c-Met protein (**A**) and mRNA (**B**) level were detected; (**C**,**D**) overexpressing miR-19a reduced c-Met protein and mRNA level. Pc9 GR cells were treated with gefitinib (1 μM) and then trasfected with miR-19a or miR-NC, c-Met protein (**C**) and mRNA (**D**) level were detected after 48 h; (**E**) miR-19a overexpression blocks the c-Met downstream pathway. Pc9 GR cells were treated with gefitinib (1 μM) and then transfected with miR-19a or miR-NC, and p-Akt and p-Erk levels were measured after 48 h; (**F**) Down-regulating miR-19a activated the c-Met downstream pathway. Pc9 cells were treated with gefitinib (0.1 μM) and then transfected with Inh-19a or Inh-NC, and the p-Akt and p-Erk levels were measured after 48 h.

c-Met plays an important role in gefitinib resistance and contributes to EMT in NSCLC cells^9, 23^. Accordingly, we found that c-Met expression negatively correlated with miR-19a expression in 5 NSCLC cell lines, as shown in Fig. 1E. Therefore, we hypothesized that miR-19a might directly target c-Met and contribute to gefitinib resistance and EMT. To test this hypothesis, we first employed The Cancer Genomic Atlas (TCGA) database, and we analyzed the relationship between miR-19a and c-Met in 449 patients with NSCLC adenocarcinoma. We found a significant inverse correlation between miR-19a and c-Met (r = −0.2113, p < 0.0001; Fig. S2A). To confirm this relationship, we examined whether miR-19a regulates c-Met expression. Our data showed that the down-regulation of miR-19a by transfecting Pc9 cells with miR-19a inhibitor significantly increased c-Met protein and mRNA expression compared with Pc9 cells transfected with inhibitor control (Fig. 4A,B). Whereas, miR-19a overexpression robustly decreased c-Met protein and mRNA expression (Fig. 4C,D). Previous studies have shown that c-Met induces gefitinib resistance and EMT by activating the AKT and ERK pathways^23^. To this end, we examined whether miR-19a regulates the c-Met/AKT and ERK pathways in gefitinib-treated cells. The activity of c-Met the pathway was reduced in cells transfected with mimic miR-19a compared with cells transfected with mimic control, as determined by the levels of phosphorylated AKT and ERK (Fig. 4E). The down-regulation of miR-19a resulted in increased levels of phosphorylated AKT and ERK, as shown in Fig. 4F. Similar data were obtained using A549 cells (Fig. S3A–F), suggesting that miR-19a affects c-Met expression and its down-stream signaling pathways.

### miR-19a regulates c-Met by targeting its 3′UTR

**Figure 5:**
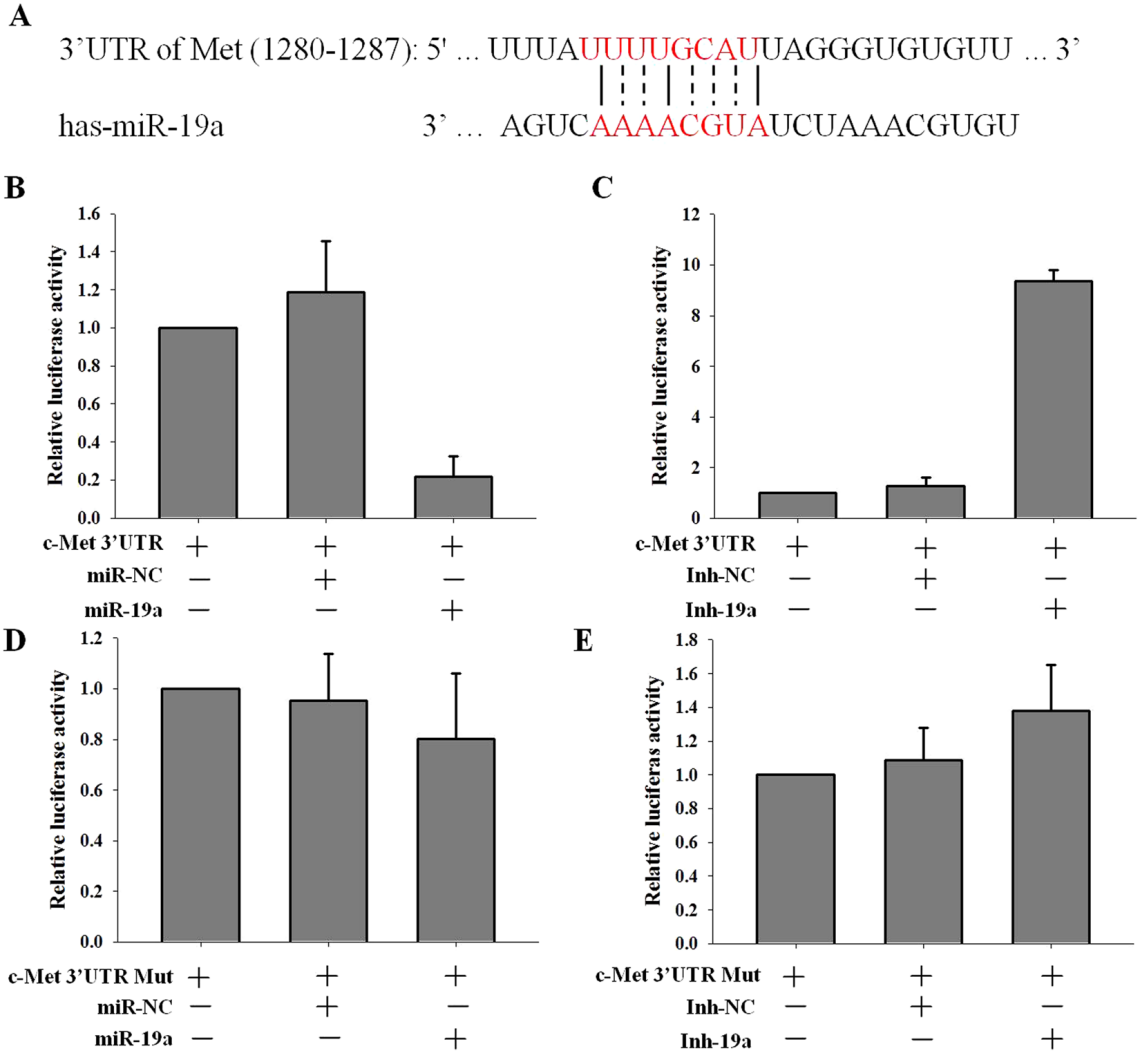
miR-19a regulates c-Met by targeting its 3′UTR. Schematic representation of the 3′UTR of c-Met with the predicted target site for miR-19a. The mutant site of c-Met 3′UTR is indicated (dotted line); (**B**,**C**) Reporter constructs containing the wild-type c-Met 3′UTR were co-transfected into Pc9 cells along with miR-19a or miR-NC (**B**) or Ihn-19a or Inh-NC (**C**); the relative luciferase activity was measured (mean ± sd, n = 3, **p < 0.01). (**D**,**E**) Reporter constructs containing the c-Met 3′UTR harboring a mutation at the predicted miR-19a target sequence were co-transfected into Pc9 cells with miR-19a or miR-NC. The same construct was co-transfected with Inh-19a or Inh-NC; the relative luciferase activity was measured (mean ± sd, n = 3, **p < 0.01).

miRNAs regulate gene expression by targeting their 3′UTR. To investigate whether miR-19a regulates c-Met expression by targeting its 3′UTR, we cloned c-Met 3′UTR sequences that contain the predicted target site (wild type) or mutated sequences (mutant type) of miR-19a into the pGL3 control vector (Fig. 5A). Our data showed that co-transfecting the 3′UTR of c-Met with miR-19a mimics significantly decreased the firefly luciferase activity of the reporter with the wild-type 3′UTR of c-Met (Fig. 5B) but did not affect the mutant reporter (Fig. 5D). Conversely, co-transfection of miR-19a inhibitor with the 3′UTR of c-Met notably increased the firefly luciferase activity of the reporter with the wild-type 3′UTR of c-Met (Fig. 5C) but not the mutant reporter (Fig. 5E). These results demonstrated that miR-19a directly targets the 3′UTR of c-Met and regulated the protein and mRNA expression of c-Met in NSCLC cells.

### miR-19a contributes to gefitinib resistance and EMT via c-Met

**Figure 6:**
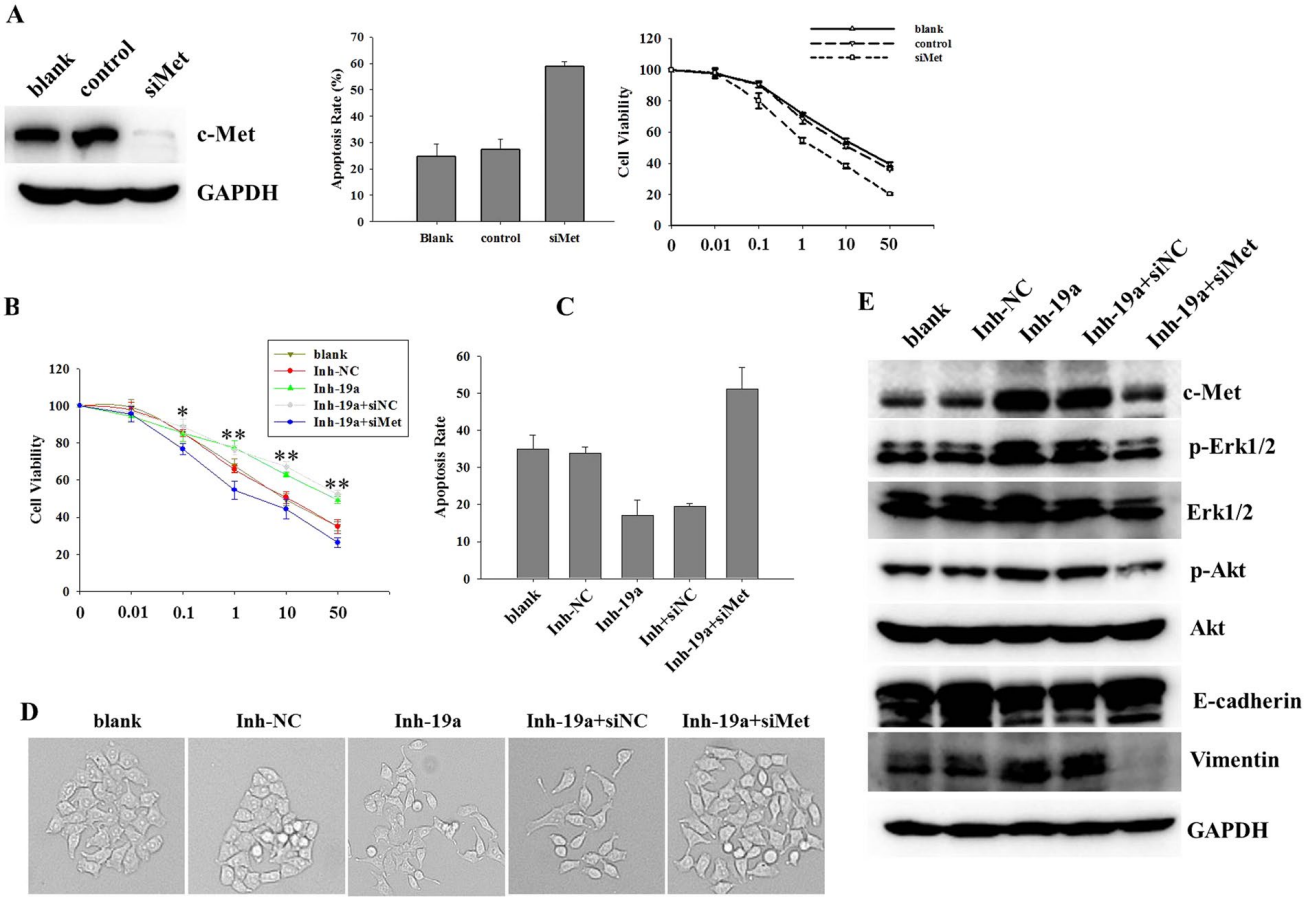
miR-19a reverses gefitinib resistance in NSCLC cells by targeting c-Met (**A**). A549 cells were transfected with c-Met siRNA (si-Met), and the c-Met protein level was detected with a western blot (left); the cells were transfected with si-Met and treated with gefitinib, and cell viability (right) and apoptosis rate (center) were measured after 48 h; (**B**) A549 cells were transfected with Inh-19a or co-transfected with Inh-19a and si-Met and treated with different doses of gefitinib for 72 h before measuring cell viability by CCK8; (**C**) The cells were transfected with Inh-19a or co-transfected with Inh-19a and si-Met and treated with 1 µM gefitinib before examining apoptosis using the Annexin V-PI assay (mean ± sd, n = 3, **p < 0.01); (**D**) The cells were transfected with Inh-19a or co-transfected with Inh-19a and si-Met; the cell morphology is shown; (**E**) The cells were transfected with Inh-19a or co-transfected with Inh-19a and si-Met, and the protein levels of c-Met and its downstream pathway effectors were detected in each group.

To further evaluate whether the effects of miR-19a on gefitinib resistance and EMT are mediated by c-Met, we first knocked down c-Met expression by transfecting small-interfering RNA (siMet) into A549 cells. Reducing c-Met expression significantly attenuated gefitinib resistance in A549 cells (Fig. 6A), which corroborated published studies. Next, we employed a “rescue” experiment by co-transfecting A549 cells with an inhibitor of miR-19a (Inh-19a) and siMet. Our data showed that the transfection of Inh-19a increased c-Met expression, and this increase was abrogated by siMet (Fig. 6E). Furthermore, the transfection of Inh-19a up-regulated c-Met and induced gefitinib resistance and EMT, but these effects were reversed when these cells were co-transfected with siMet (Fig. 6B,C and D). Finally, we evaluated c-Met-mediated downstream signaling after cells had been co-transfected with Inh-19a and siMet. Our data showed that knocking down miR-19a increased phosphorylated Akt and Erk, and these increases were offset by co-transfecting with siMet (Fig. 6E). These findings showed that the negative regulation of c-Met expression by miR-19a modulates gefitinib resistance and EMT in NSCLC cells.

### miR-19a overcomes gefitinib resistance *in vivo*

**Figure 7:**
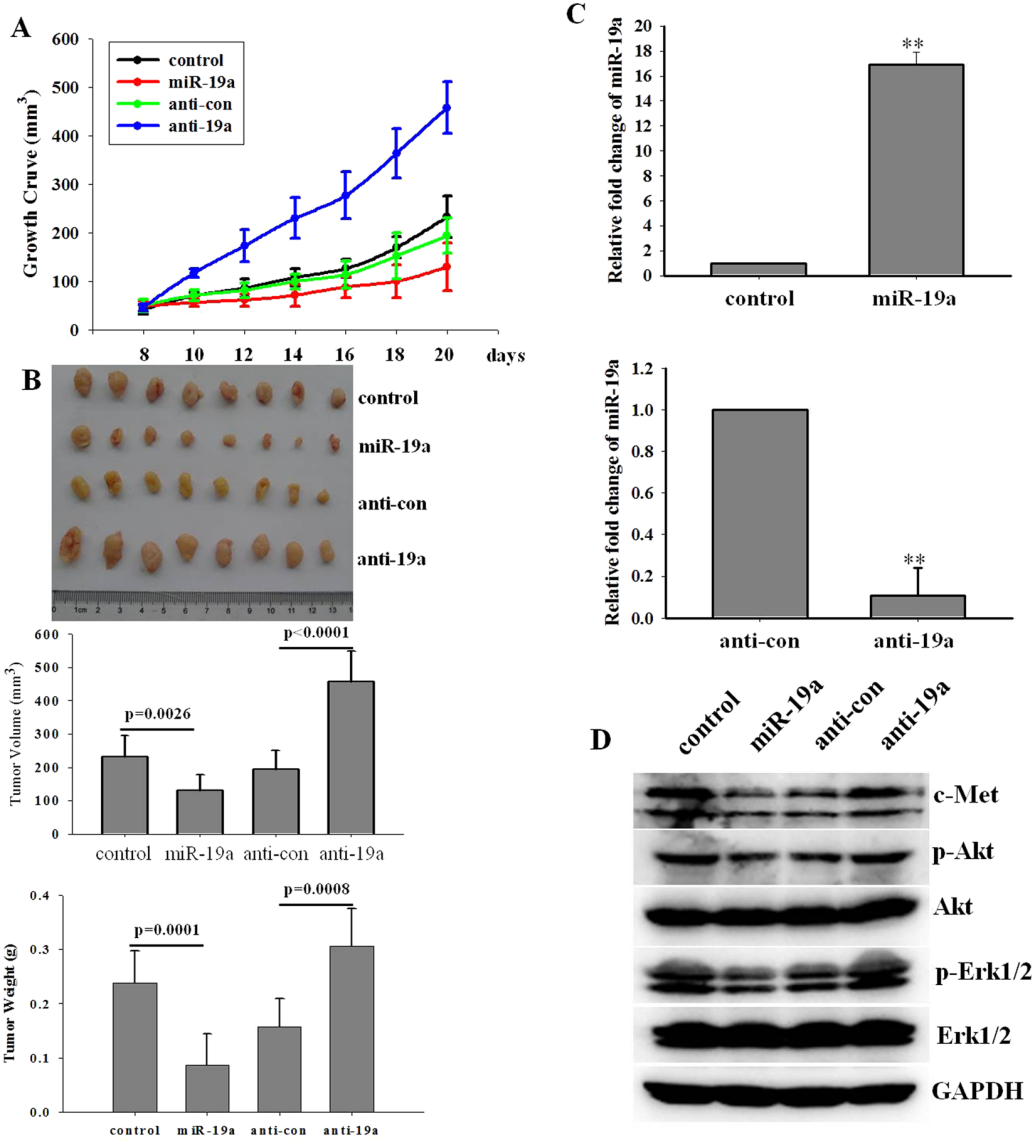
miR-19a overcomes gefitinib resistance *in vivo*. A549 cells were cultured, collected, washed and resuspended in culture medium (approximately 2 × 10^7^/ml) before being subcutaneously injected into athymic nude mice. After each tumor reached macroscopic size, gefitinib (10 mg/kg) was orally administered to each mouse every other day, and each tumor was measured. The tumor growth curves of four groups for a period of 3 weeks were generated (**A**,**B**). After 3 weeks, the mice were sacrificed, and the tumors were collected and measured; tumor in photograph (**B**, top), mean tumor volume (**B**, center), mean tumor weight (**B**, bottom) (mean ± sd, n = 8) (**C**). miR-19a expression in each group was detected by qRT-PCR; the c-Met, p-Akt, p-Erk protein levels were detected with a western blot (**D**).

To further evaluate the role of miR-19a in gefitinib resistance, we established a cell line that stably expressed high or low levels of miR-19a by infection with a lentivirus that expressed a pre-miR-19a (Lv-19a) or anti-pre-miR-19a (anti-19a) sequence in A549 cells, respectively. The cells were cultured, collected, washed and resuspended in culture medium and subcutaneously injected into athymic nude mice. All mice were sacrificed after 3 weeks, and the tumors were collected and weighed. During tumor development, the tumor volume was measured, and a growth curve was generated, as shown in Fig. 7A. After 3 weeks, the mean volume of Lv-19a tumors was significantly reduced compared with the volume of Lv-NC tumors (129.89 ± 48.86 mm^3^ versus 232.61 ± 62.72 mm^3^, respectively), whereas the mean tumor volume of anti-19a tumors was significantly increased compared with the volume of anti-NC tumors (457.10 ± 92.98 mm^3^ versus 194.15 ± 56.06 mm^3^, respectively; Fig. 7B center). We also evaluated the tumor weight and found that the mean weight of Lv-19a tumors was lower than that of Lv-NC tumors (LV-NC, 0.238 ± 0.058 g versus LV-19a, 0.086 ± 0.073 g, respectively). However, the mean weight of anti-19a tumors was higher than that of anti-NC tumors (anti-NC, 0.19 ± 0.052 g versus anti-19a, 0.357 ± 0.069 g, respectively; Fig. 7B bottom), which was consistent with the observed mean tumor volume. The qRT–PCR results confirmed that miR-19a expression was higher in the Lv-19a group than in the Lv-NC group, whereas miR-19a expression was markedly lower in the anti-19a group than in the control group (Fig. 7C). In addition, we examined the expression of c-Met in each tumor and found that c-Met expression negatively correlated with miR-19a expression in each tumor (Fig. 7D).

## Discussion

miRNAs can be oncogenic or tumor suppressive in human cancers by targeting specific genes and consequently play crucial roles in tumorigenesis and drug resistance^13, 24^. Recently, miR-19a was reported to have complex functions in multiple cancers, including NSCLC, bladder cancer, gastric and colorectal cancer, CML and hepatocellular cancer^17, 19, 25–29^. Despite the importance of miR-19a in tumorigenesis, the role of miR-19a in gefitinib resistance in NSCLC patients has not been fully investigated. In the present study, we showed that the serum expression of miR-19a was dramatically down-regulated in gefitinib-resistant NSCLC patients and cell lines compared with gefitinib-sensitive NSCLC patients and cell lines. Interestingly, miR-19a expression negatively correlated with c-Met expression. A mechanistic analysis revealed that miR-19a contributed to gefitinib resistance and EMT in NSCLC cells by targeting c-Met. Specifically, we analyzed 449 NSCLC adenocarcinoma patient samples from the TCGA database and found a negative correlation between miR-19a and c-Met, which was consistent with our observations in cell lines.

Previous study reported that miR-19a triggered EMT and promoted cell migration and invasion in lung cancer cells^17^. However, in our system, we treated NSCLC cells with gefitinib and found that overexpression miR-19a reversed EMT and prohibited cell migration and invasion (Fig. 3). Other studies found that miR-19a increased the levels of phosphorylation of Akt in breast cancer and B-cell lymphomas^30, 31^. But in our study, we found that miR-19a decreased the level of phosphorylation of Akt in NSCLC cells under gefitinib treated (Fig. 4E, Fig. S3E).The difference above may be attributed to the treatment of gefitinib and different target genes.

Currently, the detection of serum miRNA biomarkers has become one of the most important methods to assist with disease diagnosis^32, 33^. The data presented herein support that the serum level of miR-19a is down-regulated in gefitinib-resistant patients compared with gefitinib-sensitive patients. We also analyzed the ability of miR-19a expression to predict the prognosis of NSCLC patients from the TCGA database. To this end, we found that the overexpression of miR-19a predicted a slightly better prognosis, although the difference did not reach significance (Fig. S2B). In future studies, we plan to verify the ability of the serum expression level of miR-19a to predict the prognosis of patients with NSCLC treated with gefitinib in a large sample.

c-Met amplification is one of the most important mechanisms that facilitate gefitinib resistance by re-activating the p-Akt and p-Erk pathway in NSCLC^9, 34^. Although c-Met amplification plays an important role in gefitinib resistance, the mechanisms responsible for the overexpression of c-Met in NSCLC cells after gefitinib treatment are not fully understood. We found that miR-19a expression was gradually inhibited as Pc9 cells were treated with gefitinib (Fig. S2C). Furthermore, the expression of miR-19a was robustly decreased in Pc9 GR cells compared with Pc9 cells. Although, the miRnada database shows weak link between miR-19a and c-Met mRNA, we found another seed region of c-Met 3′UTR (between 1280–1287) and miR-19a as shown in Fig. 5A. Also, our data robustly supported that miR-19a significantly decreased c-Met expression (Fig. 4A) and targeted iks 3′UTR directly (Fig. 5). This study is the first to report that miR-19a can regulate c-Met protein and mRNA expression by targeting its 3′UTR, and the role of miR-19a in gefitinib resistance and EMT depends on c-Met. In conclusion, miR-19a contributes to gefitinib resistance and EMT and may serve as a biomarker of gefitinib-resistant patients. miR-19a is a potent regulator of c-Met in NSCLC cells, and introducing miR-19a into NSCLC cells may be a novel therapeutic strategy to reverse resistance to gefitinib.

## Electronic supplementary material

Supplementary information
