# Supplementary material for: miR-19a contributes to gefitinib resistance and epithelial mesenchymal transition in non-small cell lung cancer cells by targeting c-Met
Source: Sci Rep. 2017 Jun 7;7:2939. doi: 10.1038/s41598-017-01153-0 (PMC5462753; doi:10.1038/s41598-017-01153-0)
Supplement: Supplementary file 1 — Supplementary information [file 41598_2017_1153_MOESM1_ESM.docx]

**Supplementary information**

**miR-19a contributes to gefitinib resistance and epithelial mesenchymal transition in non-small cell lung cancer cells by targeting c-Met**

Xiaonian Cao^1^, Senyan Lai^2^, Fayong Hu^3^, Guodong Li^3^, Guihua Wang^2^, Xuelai Luo^2^, Xiangning Fu^1^, Junbo Hu^2^

^1^Department of Thoracic Surgery, Tongji Hospital, Tongji Medical College, Huazhong University of Science and Technology, Wuhan, 430030 China

^2^Department of Gastrointestinal Surgery Center, Tongji Hospital, Tongji Medical College , Huazhong University of Science and Technology, Wuhan, 430030, China

^3^Cancer Research Institute, Tongji Hospital, Tongji Medical College, Huazhong University of Science and Technology, Wuhan, 430030, China

**Correspondence:** Professor Junbo Hu, Cancer Research Institute, Tongji Hospital, Huazhong University of Science and Technology, Wuhan 430030, China.

**E-mail**: jbhu@tjh.tjmu.edu.cn

**Key words:** miR-19a, non-small cell lung cancer, gefitinib-resistance, c-Met

**Supplementary figures**

**Fig.S1:** **overexpression of miR-19a reverses gefitinib-resistance in A549 cells**

**
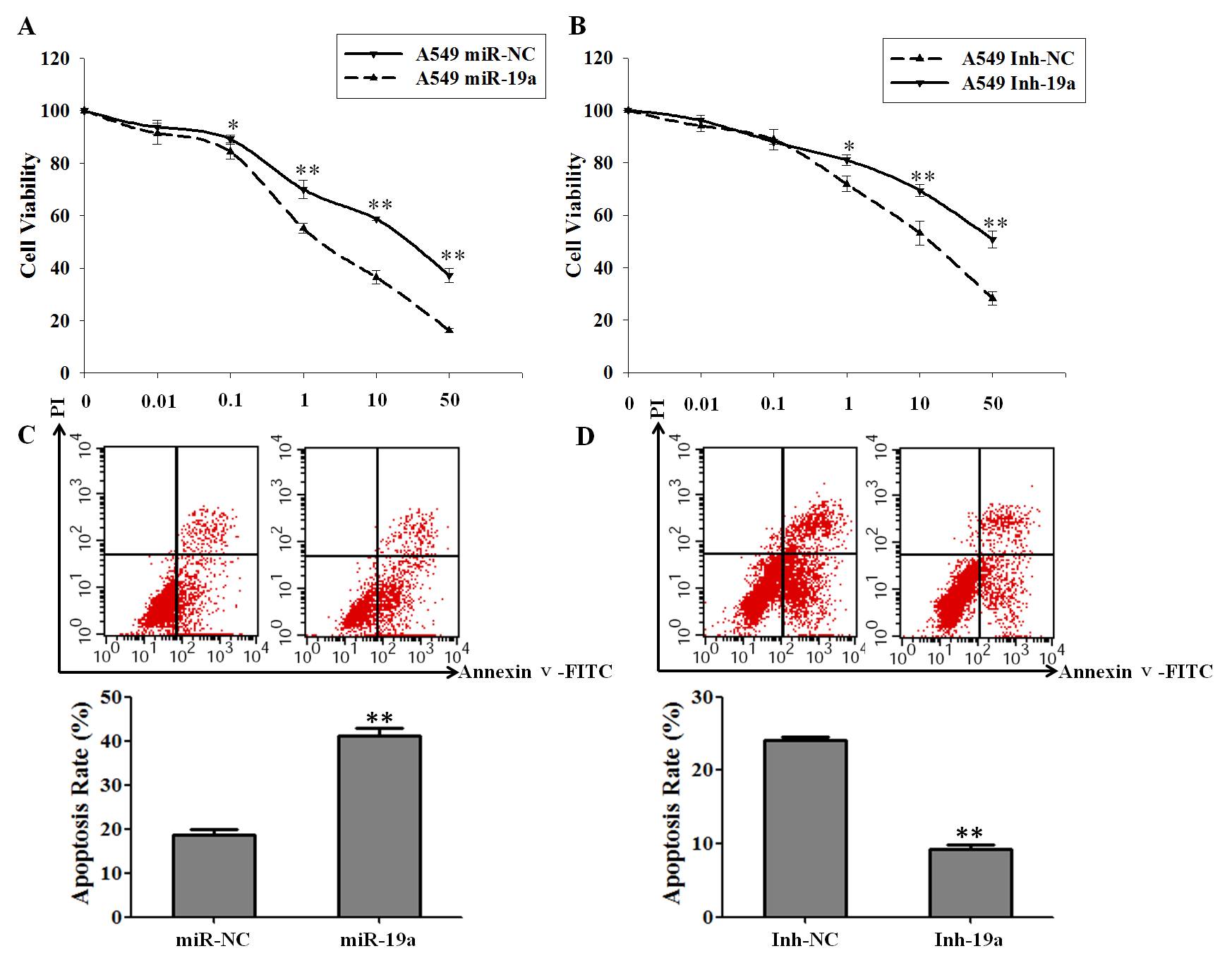
**

A. A549 (3000/plate) cells were transfected with miR-19a mimics (miR-19a) or the negative control (miR-NC) at a final concentration of 100 nM. The cells were then treated with different doses of gefitinib for 72 h, and viability was assessed using CCK8; B. A549 cells were transfected with miR-19a inhibitor (Inh-19a) or the negative control (Inh-NC), different doses of gefitinib was added and cell viability was tested by CCK8 after 72h; C. A549 cells were transfected with miR-19a or miR-NC, 5μM gefitinib was added and cell apoptosis rate was measured by Annexin V-PI after 48h. D. A549 cells were transfected with Inh-19a or Inh-NC, 5μM gefitinib was added and cell apoptosis rate was measured by Annexin V-PI after 48h. (The results above were reproducible in three independent experiments,**, p<0.01)

**Fig.S2: Correlation between miR-19a and c-Met expression in TCGA samples from patients with NSCLC**

**
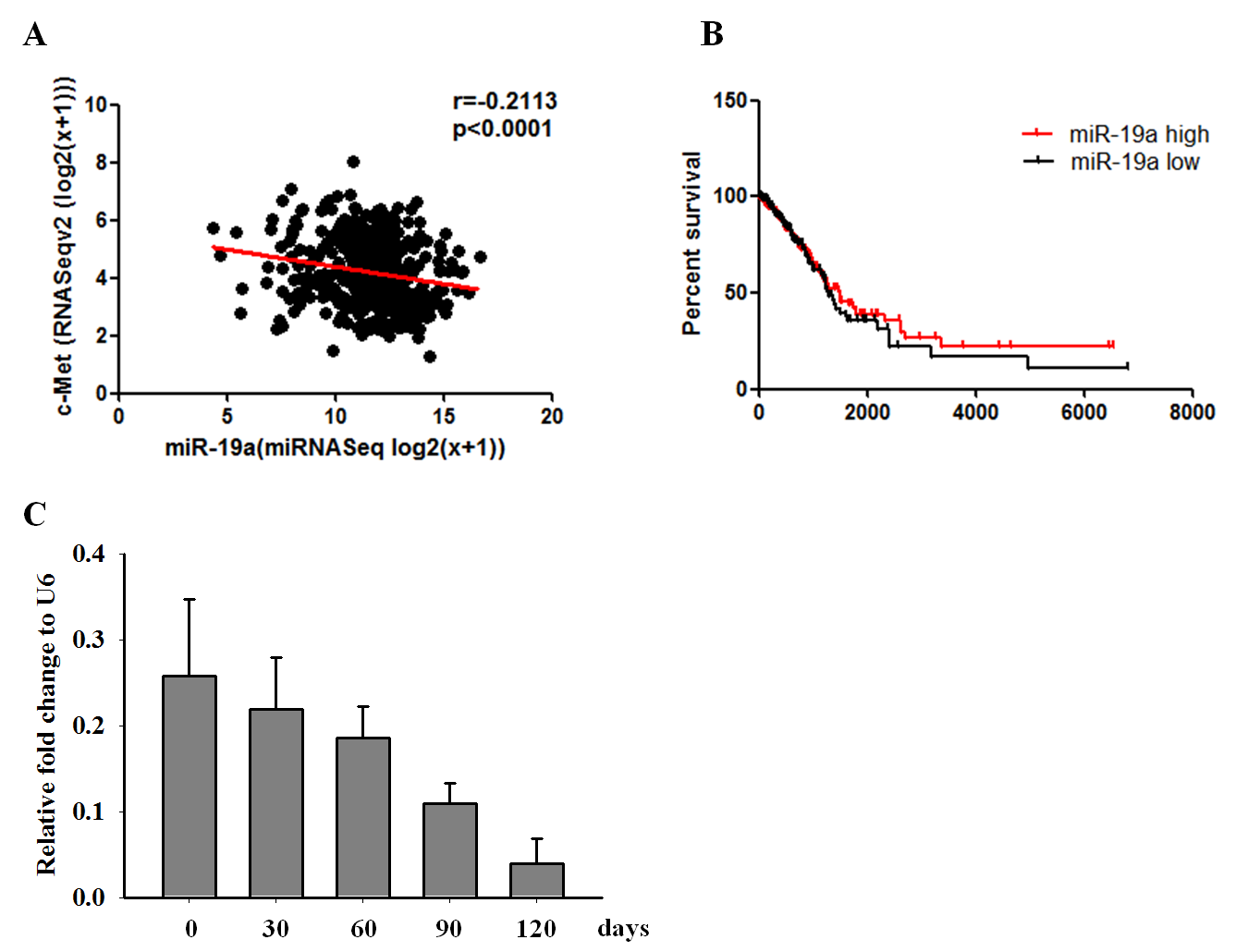
**

A. Correlation between miR-19a and c-Met mRNA expression in samples from 449 patients with NSCLC from The Cancer Genome Atlas (TCGA) (p<0.001). The Spearman’s rank-order correlation test was applied to measure the strength of the association between miR-19a and c-Met mRNA levels; B. Analyzing the correlation miR-19a expression and prognosis of patients with NSCLC from TCGA database. All TCGA data were collected and the patients who were loss of follow-up were excluded, and final 561 patients’ databases were analyzed with GraphPad Prism. *P values* were obtained for the median separate the patients in high/low (min *P value*) were recorded. C. Pc9 cells were treated with gefitinib (started at 0.1μM and increased gradually) and miR-19a expression was detected at prescribed day.

**Fig.S3: MiR-19a regulated c-Met protein and mRNA level in A549 cells**

**
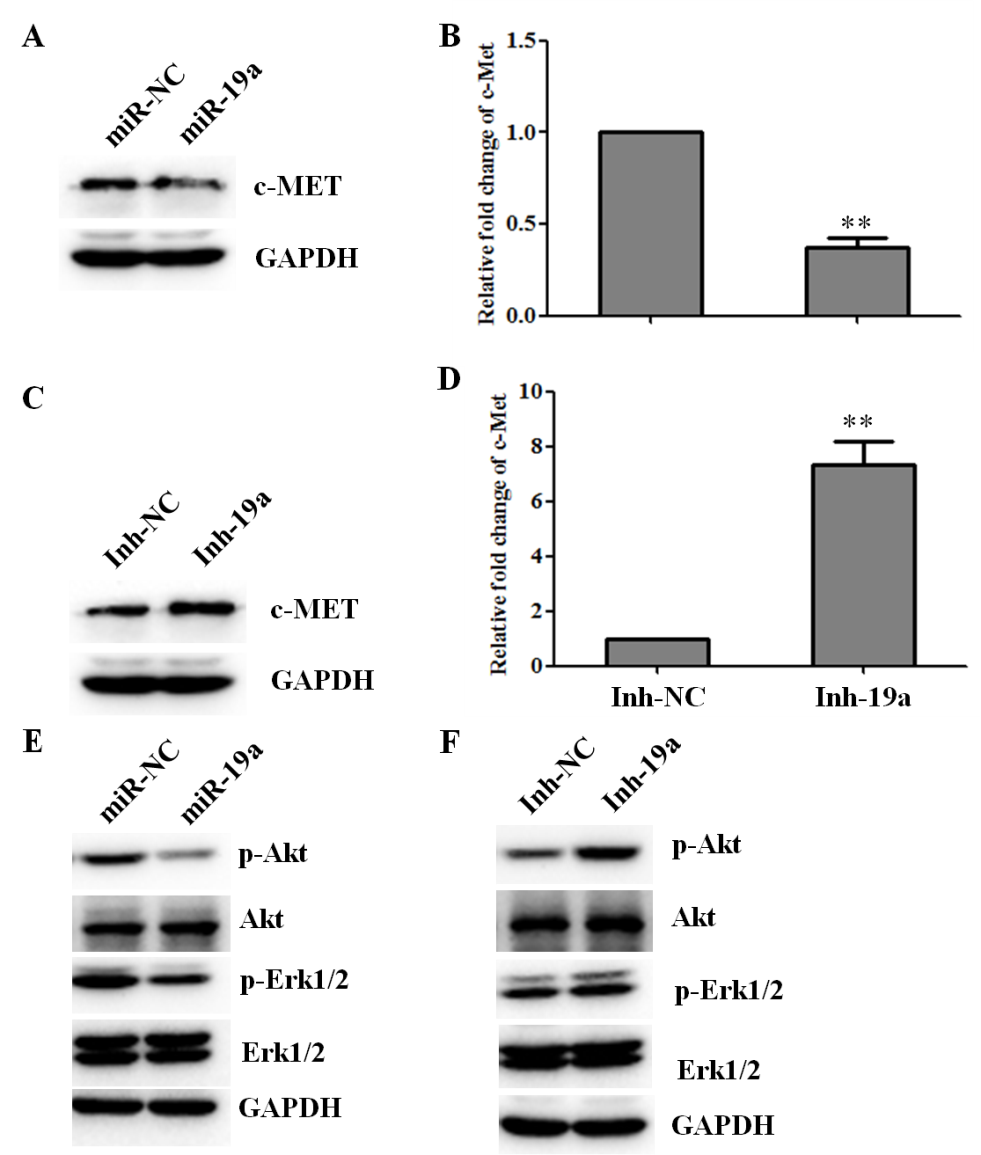
**

A, B. overexpressing miR-19a reduced c-Met protein and mRNA level. A549 cells were treated with gefitinib (1μM) and then transfected with miR-19a or miR-NC, c-Met protein (A) and mRNA (B) level were detected after 48h; C, D. down-regulated miR-19a raised c-Met protein and mRNA level. A549 cells were treated with gefitinib (1μM) and then transfected with Inh-19a or Inh-NC, after 48h, c-Met protein (C) and mRNA (D) level were detected; E. overexpression of miR-19a block c-Met downstream pathway. A549 cells were treated with gefitinib (1μM) and then transfected with miR-19a or miR-NC, p-Akt and p-Erk levels were detected after 48h; F. down-regulating miR-19a activated c-Met downstream pathway. A549 cells were treated with gefitinib (1μM) and then transfected with Inh-19a or Inh-NC, p-Akt and p-Erk levels were detected after 48h.

**Fig.S4 expression of miR-19a in NSCLC after cells were transfected with miR-19a mimics or inhibitor.**

**
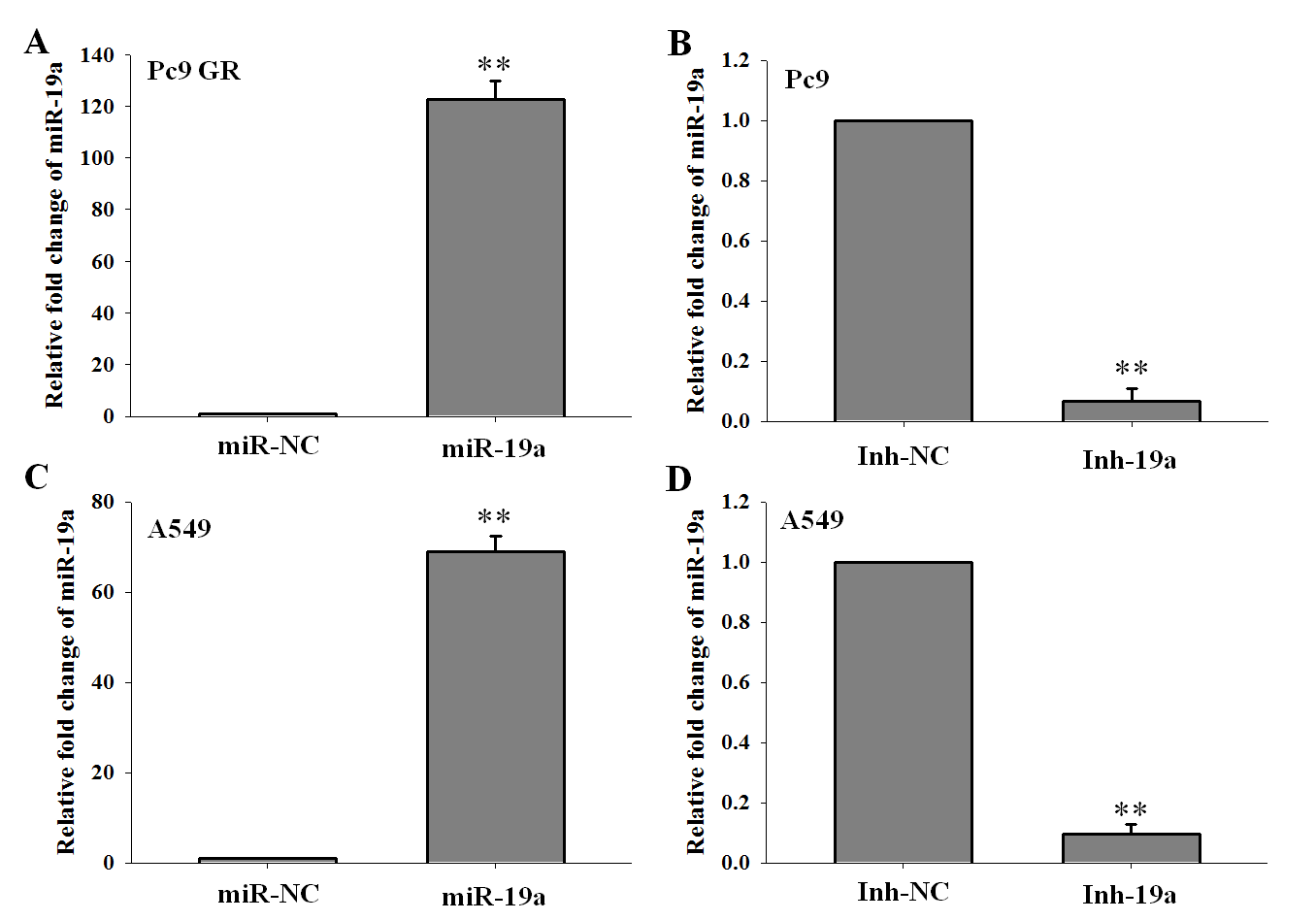
**

A. Pc9 GR cells were cultured and transfected with miR-19a mimics (miR-19a) or negative control (miR-NC) at final concentration of 100nM, cells were harvested and miR-19a was detected in each group by qRT-PCR after 48h; B. Pc9 cells were cultured and transfected with miR-19a inhibitor (Inh-19a) or negative control (Inh-NC), miR-19a expression was detected by qRT-PCR in each group; A549 cells were cultured and transfected with miR-19a (C)　or Inh-19a (D), cells were harvested and miR-19a was detected in each group by qRT-PCR. (The results above were reproducible in three independent experiments,**, p<0.01).

**Supplemental table 1**

**Primers used in this study**

|  |  | primers |
| --- | --- | --- |
| c-Met | Forward | AGCAATGGGGAGTGTAAAGAGG |
|  | Reverse | CCCAGTCTTGTACTCAGCAAC |
| c-Met 3’UTR | Forward | CGGCTAGCTGCTAGTACTATGTCAAAGCAAC |
|  | Reverse | GGCTCGAGACAAGATGTTGCATCACTTTAC |
| GAPDH | Forward | TGTGGGCATCAATGGATTTGG |
|  | Forward | ACACCATGTATTCCGGGTCAAT |
